# Presence of positive skin prick tests to inhalant allergens and markers of T2 inflammation in subjects with chronic spontaneous urticaria (CSU): a systematic literature review

**DOI:** 10.1186/s13223-020-00461-x

**Published:** 2020-08-04

**Authors:** Melanie Mitsui Wong, Paul Kevin Keith

**Affiliations:** 1 Michael G. DeGroote School of Medicine, McMaster University, Hamilton, ON Canada; 2 Department of Medicine, Division of Clinical Immunology and Allergy, McMaster University, Health Sciences Centre 3V47, 1280 Main St West, Hamilton, ON L8S 4K1 Canada

**Keywords:** Chronic spontaneous urticaria, Aeroallergen, IgE, Eosinophils, FcεR1α, Autoantibody, Anti-IgE, House dust mite, Rhinitis, T2 inflammation

## Abstract

**Background:**

Current guidelines do not recommend performing aeroallergen skin prick testing (SPT) in chronic spontaneous urticaria (CSU).

**Objective:**

The objective of this review was to investigate the presence of aeroallergen sensitization and markers of T2 inflammation in subjects with CSU.

**Methods:**

Systematic literature reviews to identify all studies that evaluated the presence of T2 markers of allergic inflammation in CSU subjects were performed.

**Results:**

In 16 studies that assessed the prevalence of positive SPT to multiple aeroallergens in CSU, 38.5% of CSU subjects had positive SPT. In three controlled studies, 34.2% of CSU subjects had positive SPT to multiple aeroallergens, compared to 13.6% of controls (p = 0.047). In 18 studies that assessed the prevalence of house dust mite (HDM) positive SPT in CSU, 27.5% of CSU subjects had positive SPT. In three controlled studies, 27.5% of CSU subjects had positive SPT to HDM, compared to 2.1% of controls (p = 0.047). Overall, CSU subjects were 3.1 times more likely to be aeroallergen-sensitized (95% CI 1.7–5.8, p = 0.0002) and 6.1 times more likely to be HDM-sensitized (95% CI 3.7–9.9, p < 0.00001) than controls. Mean total serum IgE (tIgE) levels were 238 kU/L and median tIgE levels were 164 kU/L, which was greater than the upper 90^th^ percentile of normal (< 137 kU/L). Compared to healthy controls, CSU subjects were 6.5 times more likely to have IgG autoantibody against FcεR1α (p = 0.001), 2.4 times more likely to have IgG anti-IgE antibody (p = 0.03) and 5 times more likely to have anti-thyroid peroxidase (anti-TPO) antibody (p = 0.02). When corticosteroids were withheld for ≥ 28 days, mean blood eosinophil percentage was elevated at 5.9% (normal < 4%), but other studies reporting absolute count found the mean was in the normal range, 239 $\times 10^{6} /$L (normal < 400 $\times 10^{6} /$L).

**Conclusion:**

Increased aeroallergen sensitization, tIgE, autoantibodies and blood eosinophil percentage in the CSU subjects indicates the possible importance of T2 inflammation in the pathogenesis of CSU. Further studies may be warranted to determine if specific allergen avoidance, desensitization or improvement in the mucosal allergic inflammation present in asthma and/or rhinitis has any benefit in the management of CSU.

## Background

Urticaria is a vascular reaction in the superficial skin characterized by dark-red and slightly raised wheals, that are often associated with pruritus [1]. Typically, most cases are self-limiting, benign and of short duration, with episodes rarely persisting for more than several days; however, hives and swelling can persist for weeks to years and can significantly decrease one’s quality of life. Conventionally, chronic spontaneous urticaria (CSU) is defined as continuous or recurring intermittent episodes of 6 weeks or longer [1]. The prevalence of CSU in the general population has been estimated to range from 0.5 to 5% [2]. While specific mechanisms have been implicated in the pathogenesis of CSU, autoimmunity has been suggested to be a frequent cause of the condition [3]. However, in approximately 50% of patients, its etiology remains largely unknown [4].

Skin prick testing (SPT) is a highly reliable clinical procedure used to detect the presence of IgE antibodies or sensitization to an allergen of interest and can provide clinical evidence for a diagnosis of a suspected Type 1 Immunoglobulin E (IgE)-dependent allergic disease. While the potential role of immediate hypersensitivity in the pathogenesis of CSU has been considered in only a few reports, current guidelines state that “rarely, IgE-mediated reactions from foods, drugs, or other allergens might result in CSU” and that “Immediate hypersensitivity skin or serologic testing for food or other allergens is rarely useful and not recommended on a routine basis.” [2] SPT is not felt to be relevant, as international guidelines state “Type I allergy is an extremely rare cause of CSU” [5].

However, omalizumab (anti-IgE) has been shown to be an effective therapy for CSU, suggesting the importance of IgE in this condition. The presence of immunoglobulin G (IgG) autoantibody to the high-affinity Fc region of IgE (FcεR1α) in some subjects has been proposed as a possible cause of CSU, however autoantibodies may be more commonly found when atopy is present [6–9]. Other autoantibodies including IgG anti-IgE and anti-thyroid peroxidase (anti TPO) have also been described [10, 11]. A group in New York, USA found that 67% of CSU patients with allergic rhinitis in their clinic were sensitized to mugwort compared to 31% positive SPT in subjects with allergic rhinitis alone (p = 0.004) [12]. They speculated that cross reactivity to foods and spices may play a role in CSU, but didn’t consider the effects of inhaled mugwort or other aeroallergens as a possible cause. Recently, it was reported that following house dust mite (HDM) immunotherapy treatment in two patients with persistent HDM allergic rhinitis (AR) and CSU, both their nasal and CSU symptoms significantly improved [13]. In addition, there are case reports of three different anti-IL5 monoclonal antibodies [14–16] as well as a case series of the anti-IL4 and anti-IL13 monoclonal antibody, dupilumab [17] improving symptoms of CSU in subjects with severe eosinophilic asthma. Recently, a CSU cohort was found to have IgE against staphylococcal enterotoxin B (SEB), commonly found in subjects with chronic rhinosinusitis with nasal polyposis (CRSwNP) [18]. SEB IgE level was positively correlated with CSU disease activity, elevated total serum IgE (tIgE) and increased basophil histamine-release.

Here, we systematically evaluated the literature for the presence of T2 markers of allergic inflammation in CSU subjects, specifically the presence of sensitization to aeroallergens in CSU, the presence of IgG autoantibody to FcεR1α and other markers of T2 inflammation including tIgE, blood eosinophil count and concomitant AR and asthma.

## Methods

### Methods 1.1

A search for all original studies referring to the presence of positive SPT to inhalant allergens in subjects > 18 years old with CSU was conducted following the Preferred Reporting Items for Systematic Reviews and Meta-Analyses (PRISMA) guidelines for reporting. We performed electronic searches within the PubMed, Ovid Medline, EMBASE and Cochrane Database of Systematic Reviews and Cochrane Controlled Register of Trials (CENTRAL) databases for English language articles published in peer-reviewed journals up to May 2020. The key words and search terms were as follows: (chronic urticaria OR chronic hives OR chronic spontaneous urticaria OR chronic idiopathic urticaria) AND (inhalant allergen* OR aeroallergen* OR allergen* OR mite* OR pollen OR skin prick test*). Manual searches of the Annals of Allergy journals, which were not available in electronic databases were also executed. Reference lists of identified articles were scanned to collect additional records.

### Methods 1.2

A search for all original studies referring to the levels of tIgE in adolescent and adult subjects with CSU treated with omalizumab was conducted following the PRISMA guidelines for reporting. We performed electronic searches within the PubMed, Ovid Medline and CENTRAL databases for English language articles published in peer-reviewed journals up to May 2020. The key words and search terms were as follows: (chronic urticaria OR chronic hives OR chronic spontaneous urticaria OR chronic idiopathic urticaria) AND (omalizumab). Reference lists of identified articles were scanned to collect additional records.

### Methods 1.3

A search for all original studies assessing the presence of IgG autoantibody to FcεR1α, IgG anti-IgE antibody and anti-TPO antibody in patients with CSU was also conducted following the PRISMA guidelines for reporting. We performed electronic searches within the PubMed, Ovid Medline and CENTRAL databases for English language articles published in peer-reviewed journals up to May 2020. The key words and search terms were as follows: (chronic urticaria OR chronic hives OR chronic spontaneous urticaria OR chronic idiopathic urticaria) AND (autoantibod* OR FcepsilonRI OR FcεR1α OR high-affinity IgE receptor).

### Methods 1.4

A search for all original studies assessing the total eosinophil percentage and/or absolute eosinophil counts in adolescent and adult subjects with CSU was conducted following the PRISMA guidelines for reporting. We performed electronic searches within the PubMed, Ovid Medline and CENTRAL databases for English language articles published in peer-reviewed journals up to May 2020. The key words and search terms were as follows: (chronic urticaria OR chronic hives OR chronic spontaneous urticaria OR chronic idiopathic urticaria) AND (eosino*). Reference lists of identified articles were scanned to collect additional records.

#### Eligibility criteria

The search was limited to studies that met the following pre-defined inclusion criteria:

##### Methods 1.1

1. Participants: Male or female subjects with CSU who underwent testing for aeroallergen sensitization (subjects > 18 years of age).
2. Intervention: Male or female subjects diagnosed with CSU by explicit diagnostic criteria.
3. Control: Healthy male or female subjects with no history of atopy.
4. Outcomes: A measure of SPT results.
5. Types of studies: Any observational study including case control trials, retrospective chart reviews, cross-sectional studies, and case series.

##### Methods 1.2

1. Participants: Male or female subjects with CSU who underwent testing for tIgE levels (subjects > 18 years of age).
2. Intervention: Male or female subjects diagnosed with CSU by explicit diagnostic criteria.
3. Control: Healthy male or female subjects with no history of atopy.
4. Outcomes: A measure of tIgE levels.
5. Types of studies: Any observational study including case control trials, cross-sectional studies, and case series.

##### Methods 1.3

1. Participants: Male or female subjects with CSU who underwent testing for IgG autoantibody to FcεR1α (subjects > 18 years of age).
2. Intervention: Male or female subjects diagnosed with CSU by explicit diagnostic criteria.
3. Control: Healthy male or female subjects with no history of atopy.
4. Outcomes: A measure of IgG autoantibody to FcεR1α IgG anti-IgE antibody and anti- TPO antibody.
5. Types of studies: Any observational study including case control trials, cross-sectional studies, and case series.

##### Methods 1.4

1. Participants: Male or female subjects with CSU who underwent testing for total eosinophil percentage and/or absolute eosinophil counts (pediatric and adult subjects).
2. Intervention: Male or female subjects diagnosed with CSU by explicit diagnostic criteria.
3. Control: Healthy male or female subjects with no history of atopy.
4. Outcomes: A measure of total eosinophil percentage and/or absolute eosinophil counts.
5. Types of studies: Any observational study including case control trials, cross-sectional studies, retrospective chart reviews and case series.

Articles describing acute urticaria or inducible urticaria were excluded. Non-full text publications, letters to the editor, case reports, as well as articles that were not published in the English language were also excluded. Finally, abstract-only publications were not considered due to lack of detailed raw data.

#### Data collection and quality assessment

Two reviewers independently assessed the titles and abstracts of all retrieved publications to identify eligible and relevant studies. Full texts of selected publications were then retrieved if necessary and eligibility criteria was considered. Any discrepancies that were raised during data collection were resolved through consensus. Should the publication have met all eligibility criteria, information regarding the first author, year of publication, country that the study was conducted in, definition of CSU, indication of treatment provided, and the:

- 1.1: CSU population who underwent skin prick testing, number of inhalant allergens tested, SPT results.
- 1.2: CSU population who underwent IgE testing and the resulting mean tIgE levels.
- 1.3. CSU population who underwent testing for IgG autoantibody to FcεR1α, IgG anti-IgE antibody and anti-TPO antibody.
- 1.4: Mean and median total eosinophil levels as well as mean and median absolute eosinophil counts, was extracted.

#### Effect measures

Forest plots were used to assess the association of CSU with positive SPT to aeroallergens including HDM, as well as with the presence of IgG autoantibody to FcεR1α, IgG anti-IgE antibody and anti-TPO antibody. We used Review Manager version 5.3.4 to calculate mean differences and risk ratios with associated 95% CIs for dichotomous outcomes. The Mantel–Haenszel approach was chosen by using a random-effects model according to methods suggested in the Cochrane Handbook for Systematic Reviews of Interventions.

## Results

**Fig. 1:**
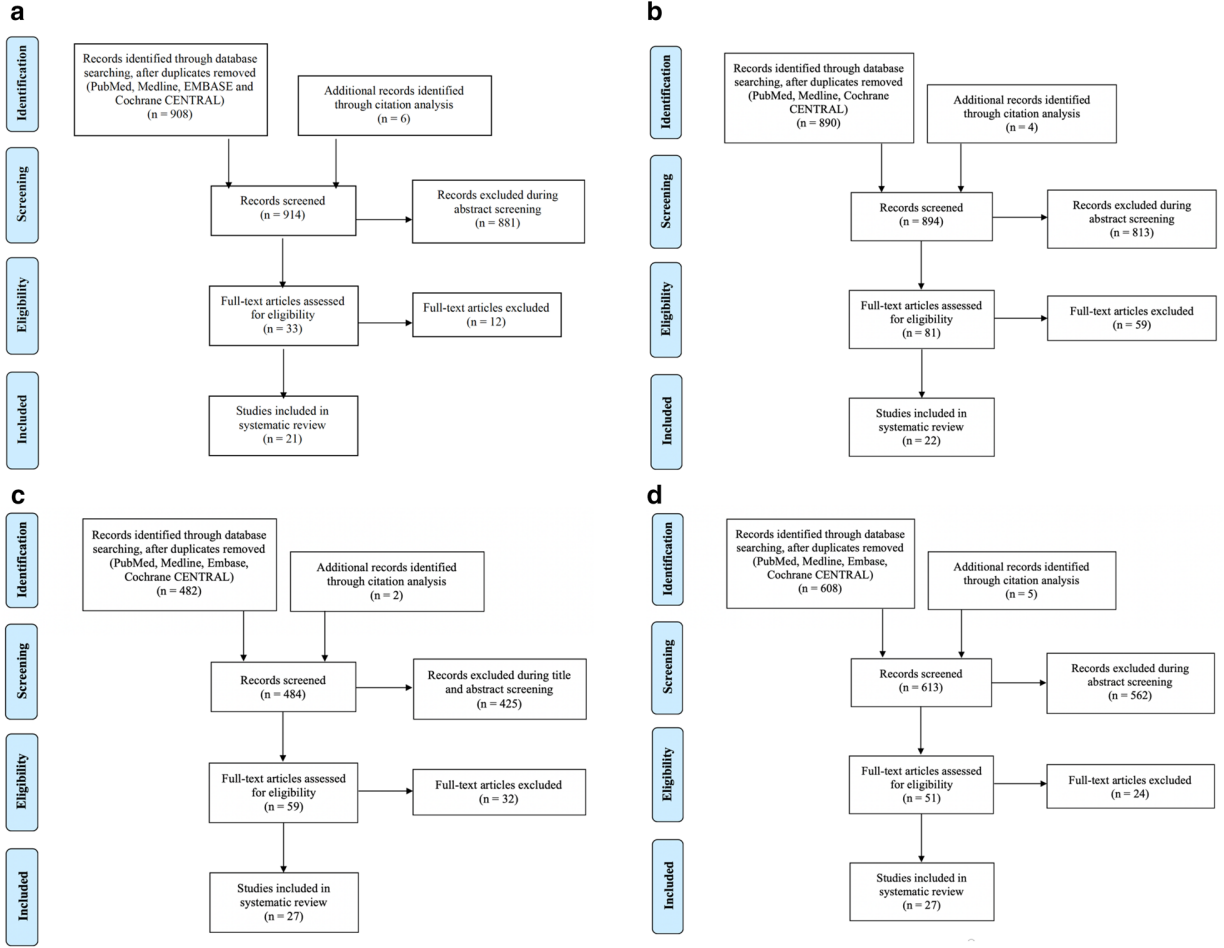
**a** PRISMA flow diagram of literature review process for the presence of aeroallergen positive SPT in CSU subjects. **b** PRISMA flow diagram of literature review process for total IgE levels in CSU subjects. **c** PRISMA flow diagram of literature review process for autoantibodies in CSU subjects. **d** PRISMA flow diagram of literature review process for total eosinophil percentage and/or absolute eosinophil counts in CSU subjects

To identify the presence of positive SPT to aeroallergens in CSU subjects, 908 publications were retrieved from database searches following duplicate removal, as well as an additional 6 publications identified through citation analysis. 33 were screened for full-text review. Of these, 12 did not meet the outlined eligibility criteria, yielding 21 full texts from 11 countries involving a total of 2982 CSU subjects that were included for analysis (Fig. 1a).

**Fig. 2:**
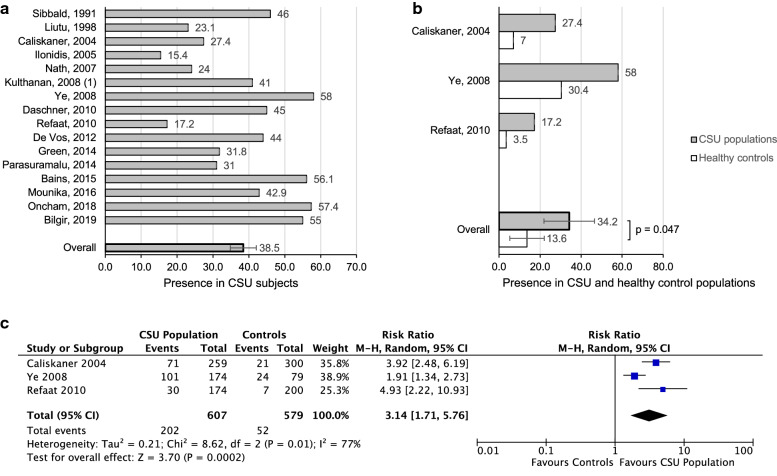
**a** Presence of positive SPT to multiple or single aeroallergens in CSU populations in 16 studies. **b** Presence of positive SPT to multiple or single antigens in CSU populations in 3 studies, compared to healthy control groups with no history of atopy (p = 0.047). **c** Forest plot graphic assessing the relative risk of positive SPT to multiple or single aeroallergens. M–H, Mantel–Haenszel approach

16 studies focused on multiple allergen extracts, namely pollens, animal dander and moulds (Table 1). In addition, some studies [12, 19–21] did not report mean numbers of positive SPTs; however, they indicated what percentage of participants were allergic to at least one antigen. As such, we included these values at the bottom of Table 1. As shown in Fig. 2a, the presence of multiple aeroallergen positive SPT in all study groups was 38.5% (standard error of the mean [SEM] 3.6).

**Table 1:** Summary of all studies measuring multiple aeroallergen SPT in subjects with CSU

Reference (author, year)	Country	Definition of CSU in weeks	Number of inhalant allergens tested	Number of CSU subjects undergoing SPT	% of positive SPT for aeroallergens in CSU patients	Number of healthy controls undergoing SPT	% of positive SPT for aeroallergens in controls	Number of antihistamine (AH) and/or systemic corticosteroid (SC)-free days	% of CSU subjects with atopic diseases: allergic rhinitis (AR) and/or asthma (A)	Total IgE levels in CSU subjects
Sibbald, 1991 [53]	Canada	–	12	75	46	–	–	–	10 (A)	23% > 200 IU/L
Liutu, 1998 [54]	Finland	> 8	≥ 4	91	23.1	–	–	–	–	49.3% > 111 IU/L
Caliskaner, 2004 [51]	Turkey	> 6	56	259	27.4	300	7	7 (AH)	Excluded AR/A	–
Ilonidis, 2005 [55]	Greece	–	8	130	15.4	–	–	–	–	–
Kulthanan, 2008 [56]	Thailand	> 6	12	88	41	–	–	3 (AH) 28 (SC)	20.5 (AR) 6.8 (A)	–
Ye, 2008 [57]	Korea	> 6	50	174	58.0	79	30.4	3 (AH) 3 (SC)	36.1 (AR) 7.7 (A)	–
Daschner, 2010 [58]	Spain	–	10	80	45	522 (with AR/A)	–	–		
Refaat, 2010 [59]	Egypt	–	9	174	17.2	200	3.5	5 (AH)	Excluded AR/A	–
Green, 2014 [60]	Canada	–	30	44	31.8	–	–	–	–	–
Bains, 2015 [52]	India	–	66	41	56.1	–	–	3 (AH) 14 (SC)	Excluded AR/A	–
Oncham, 2018 [61]	Thailand	–	17	140	57.4	–	–	–	–	–
Bilgir, 2019 [62]	Turkey	> 6	–	302	55	–	–	–	–	–
Nath, 2007 [19]	India	> 6	11	50	≥ 24	–	–	–	–	–
De Vos, 2012 [12]	United States	> 6	22	19	≥ 44	19	≥26	–	Excluded AR	–
Parasuramalu, 2014 [20]	India	> 6	26	300	1	–	–	15 (AH)	11.3 (AR) 2.3 (A)	–
Mounika, 2017 [21, 63]	India	> 6	40	56	≥ 42.9	–	–	2 (AH) 14 (SC)	–	–

*AH* antihistamine, *SC* systemic corticosteroid, *AR* allergic rhinitis, *A* asthma

Only three studies (607 CSU participants) that investigated the association of CSU with positive SPT for multiple aeroallergens included a healthy control population with no history of atopy. In each of the three publications, the CSU group had a higher percentage of positive SPT to aeroallergens compared to the control group, as shown in Fig. 2b (p = 0.047). On average, 34.2% of the total study population had positive skin tests to aeroallergens (SEM 12.3) while a review of control groups revealed a presence of 13.6% (SEM 8.4). Due to the lack of a control group in the remaining studies, it becomes more difficult to determine if the increased percentage of positive SPT results are due to a diagnosis of CSU or a result of other factors. Combined relative risk (RR) was 3.1 (95% CI 1.7–5.8) with significant heterogeneity (p = 0.0002; I^2^ = 77%) (Fig. 2c). Overall, the final estimate from the forest plots revealed an increased risk of positive SPT to aeroallergens in subjects with CSU.

**Fig. 3:**
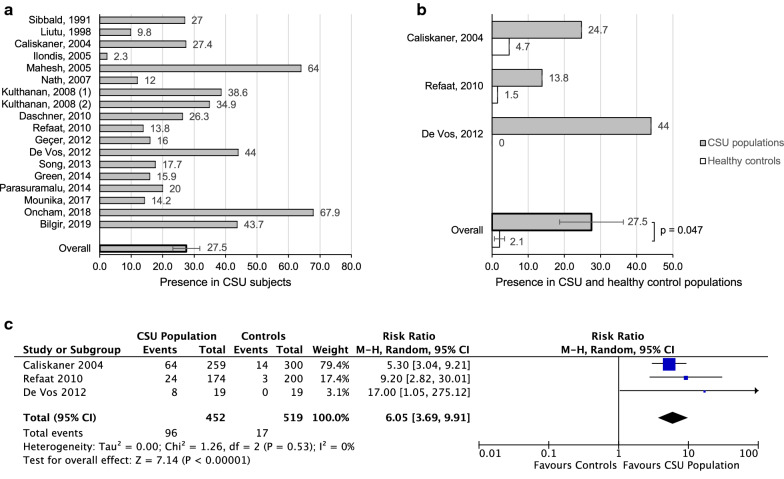
**a** Presence of positive SPT to house dust mite antigen in CSU subjects in 18 studies. **b** Presence of positive SPT to house dust mite antigen in CSU populations in 3 studies, compared to healthy control groups with no history of atopy (p = 0.047). **c** Forest plot graphic assessing the relative risk of positive SPT to house dust mite antigen in CSU populations compared to healthy controls,  M-H, Mantel–Haenszel approach

The most frequently assessed allergen was HDM, which was highlighted in 18 studies (Table 2). The presence of HDM positive SPT in all 18 study groups was 27.5% (SEM 4.3) (Fig. 3a). Only three studies (452 CSU participants) investigated the association of HDM positive SPT in both CSU and healthy control populations with no history of atopy. On average, 27.5% of the study population (SEM 8.8) were sensitive to HDM, while only 2.1% of the control group (SEM 1.4) tested positive to HDM (p = 0.047) (Fig. 3b). Results reveal a combined RR of 6.1 (95% CI 3.7–9.9) with no heterogeneity (p < 0.00001; I^2^ = 0%), as shown in Fig. 3c. Overall, the final estimates revealed an increased risk of positive SPT to HDM in subjects with CSU. Mahesh et al. found a high number of positive HDM results in control subjects, 23%, but did not read skin tests in a standard fashion, as the authors interpreted a positive skin test as 50% or more compared to the histamine test. As such, the data from this study was not included in Fig. 3b or c. In the Nath et al. study, only 12% patients had positive SPT to HDM, but they only tested one HDM, *Dermatophagoides farinae*.

**Table 2:** Summary of studies performing house dust mite allergen SPT in subjects with CSU

Reference (author, year)	Region	Definition of CSU in weeks	Number of CSU subjects undergoing SPT	% of positive SPT for House Dust Mite in Study Group	Number of healthy controls undergoing SPT	% of positive SPT for House Dust Mite in Control Group	Number of antihistamine (AH) and/or systemic corticosteroid (SC)-free days	% of subjects with atopic diseases: allergic rhinitis (AR) and/or asthma (A)	Total IgE levels in CSU subjects
Sibbald, 1991 [53]	Canada	–	75	27	–	–	–	–	23% > 200 IU/L
Liutu, 1998 [54]	Finland	> 8	91	9.8	–	–	–	–	49.3% > 111 IU/L
Caliskaner, 2004 [51]	Turkey	> 6	259	24.7	300	4.7	7 (AH)	Excluded AR/A	–
Ilonidis, 2005 [55]	Greece	–	130	> 2.3	–	–	–	–	–
Mahesh, 2005 [64]	India	> 6	122	64	25	28	–	28.7 (AR) 23.8 (A)	–
Nath, 2007 [19]	India	> 6	50	12 (D. farinae only)	–	–	–	–	–
Kulthanan, 2008 [56]	Thailand	> 6	88	38.6	–	–	3 (AH) 28 (SC)	20.5 (AR) 6.8 (A)	–
Kulthanan, 2008 [65]	Thailand	> 6	172	34.9	–	–	3 (AH) 28 (SC)	20.4 (AR) 4.7 (A)	–
Daschner, 2010 [58]	Spain	–	80	26.3	522 (with AR/A)	–	–	–	–
Refaat, 2010 [59]	Egypt	–	174	13.8	200	1.5	5 (AH)	Excluded AR/A	–
Geçer, 2012 [66]	Turkey	–	50	16	–	–	–	–	–
De Vos, 2012 [12]	United States	> 6	19	44	19	0	–	Excluded AR	–
Song, 2013 [67]	China	> 6	862	17.7	–	–	–	4.5 (AR) 1.7 (A)	–
Green, 2014 [60]	Canada	–	44	15.9	–	–	–	–	
Parasuramalu, 2014 [20]	India	> 6	300	20	–	–	15 (AH)	11.3 (AR) 2.3 (A)	–
Mounika, 2017 [63]	India	> 6	56	14.2	–	–	–	–	–
Oncham, 2018 [61]	Thailand	–	140	67.9	–	–	–	–	–
Bilgir, 2019 [62]	Turkey	> 6	302	43.7	–	–	–	–	–

**Fig. 4:**
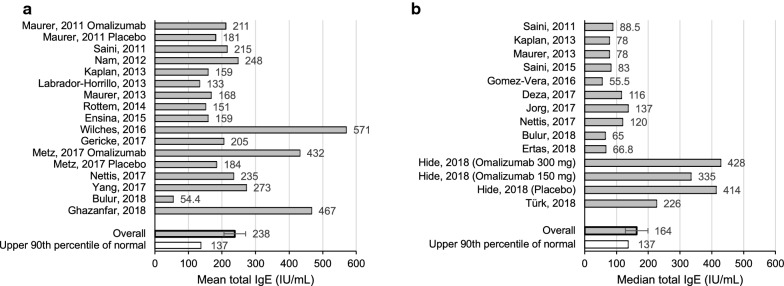
**a** Mean total IgE in CSU populations undergoing treatment with omalizumab from 15 studies, compared to the upper limit (90th percentile) of normal total IgE. **b** Median total IgE in CSU populations undergoing treatment with omalizumab or placebo from 12 studies

In identifying studies that evaluated mean tIgE levels in CSU subjects undergoing treatment with omalizumab, from 890 publications retrieved from database searches following duplicate removal, as well as an additional 4 publications identified through citation analysis, 81 were screened for full-text review. Of these, 59 did not meet the outlined eligibility criteria, yielding 22 full texts that were included for analysis (Fig. 1b). Of the 22 studies, 15 provided the mean tIgE levels in the CSU population undergoing treatment with omalizumab, with absolute values ranging from 0 to 5600 kU/L (Table 3). A recent study by Omenaas et al. reveals the 90th percentile of the distribution of total IgE in a general population was 137 kU/L [22]. While a number of studies have attempted to quantitate tIgE in a general population, these studies may have used different methodologies in their analyses and thus, comparisons should be made with caution. An average of these results revealed an overall mean total of 238 kU/L (SEM 32.1), compared to 137 kU/L, the upper 90th percentile of a general population, kU/L (Fig. 4a) and an overall median total of 164 kU/L (SEM 35.4) (Fig. 4b).

**Table 3:** Summary of studies showing mean and median total IgE levels in subjects with CSU undergoing treatment with omalizumab or placebo

Reference (author, year)	Region	Definition of CSU in weeks	Treatment	Number of CSU subjects undergoing IgE testing	Mean total IgE (kU/L) (SD)	Median Total IgE (kU/L) (IQR)	% of subjects previously on systemic corticosteroids (SC)	Number of antihistamine (AH) or systemic corticosteroid-free days	Range (kU/L)
Maurer, 2011 [68]	Germany	> 6	Omalizumab	27	211 (158)	–	–	–	–
			Placebo	22	181 (136)	–	–	–	–
Saini, 2011 [69]	USA and Germany	> 6	Omalizumab or Placebo	90	215 (432)	88.5	–	–	2–3510
Nam, 2012 [70]	Korea	> 6	Omalizumab	26	248 (275)	–	42.3	–	–
Kaplan, 2013 [71]	Various countries	> 6	Omalizumab or Placebo	335	159 (288)	78	57.9	16	1–3050
Labrador-Horrillo, 2013 [72]	Spain	> 6	Omalizumab	107	133	–	77.3	–	2–1042
Maurer, 2013 [73]	Various countries	> 6	Omalizumab or Placebo	322	168 (232)	78	–	30 (SC)	–
Rottem 2014 [74]	Israel	> 6	Omalizumab	43	151 (255)	–	88	–	–
Saini, 2015 [35]	Various countries	> 6	Omalizumab or Placebo	318	–	83	–	16 (SC)	1–5000
Ensina, 2016 [75]	Brazil	–	Omalizumab	27	159	–	–	–	0.2–774
Gomez-Vera, 2016 [25]	Mexico	> 6	Omalizumab	26	–	55.5	–	–	31.5–186
Wilches, 2016 [76]	Ecuador	> 6	Omalizumab	26	571	–	–	–	55–2500
Deza 2017 [77]	Spain	> 6	Omalizumab	47	–	116	–	–	42–277
Gericke, 2017 [78]	Germany	> 6	Omalizumab	56	205 (230)	–	–	–	–
Jorg, 2017 [79]	Switzerland	> 6	Omalizumab or Placebo	30	–	137 (37.8–311)	–	AH, SC allowed	–
Metz, 2017 [80]	Germany	> 6	Omalizumab	20	432 (1031)	–	–	3 (AH), 30 (SC)	–
			Placebo	10	184 (242)	–	–	3 (AH), 30 (SC)	–
Nettis, 2017 [81]	Italy	> 6	Omalizumab	290	235 (526)	120	73.5	Allowed (AH, SC)	0–5237
Yang, 2017 [82]	Taiwan	> 6	Omalizumab	17	272.6	–	23.5	–	8.9–1510
Bulur, 2018 [83]	Turkey	> 6	Omalizumab	132	54.4 (150)	65	62.1	Allowed (AH, SC)	7–978.9
Ertas, 2018 [84]	Turkey	> 6	Omalizumab	96	–	66.8	–	–	20.4–127
Ghazanfar, 2018 [85]	Denmark	> 6	Omalizumab	117	467 (1076)	–	–	Allowed (AH)	–
Hide, 2018 [86]	Japan	> 6	Omalizumab 150 mg	71	–	335	26.5	30	15–2360
			Omalizumab 300 mg	73	–	428	37.1		20–4950
			Placebo	73	–	414	36.1		0–5600
Türk, 2018 [87]	Turkey	> 6	Omalizumab	25	–	226	–	–	56–340

*AH* antihistamine, *SC* systemic corticosteroids

To identify studies evaluating the association between the presence of IgG autoantibody to FcεR1α (Table 4), IgG anti-IgE antibody (Table 5) and anti-TPO antibody (Table 6) in CSU subjects and healthy controls, 482 papers were retrieved from database searches following duplicate removal. 57 full texts were reviewed including an additional two papers from citation analysis, and 27 publications were accepted (Fig. 1C).

**Table 4:** Summary of studies detecting IgG autoantibody to FcεR1α in subjects with CSU

Reference (author, year)	Region	Definition of CSU in weeks	Number of CSU patients undergoing testing	Number of healthy controls undergoing testing	% prevalence of IgG autoantibody to FcεR1α in study group	Functional assay (F) or non-functional assay (N)	% prevalence of IgG autoantibody to FcεR1α in control group	Number of antihistamine (AH) and/or systemic corticosteroid (SC)-free days
Hide, 1993 [88]	England	> 8	26 + ASST	10	46.1	F	0	2 (AH)
Fiebiger, 1995 [11]	Austria	> 8	32	15	37	N	0	Not stated
Zweiman, 1996 [89]	United States	–	70	20	30	F	15	Not stated
Ferrer 1998 [10]	United States	> 12	53	24	64	N	0	2 (AH)
Fiebiger, 1998 [90]	Austria	> 8	281	41	38	N	–	Not stated
Zuberbier, 2000 [91]	Austria	> 6	48	5	35	N	0	Not stated
Sabroe, 2002 [92]	England	> 6	78	39	26 15	F N	0	3 (AH), 28 (SC)
Hidvegi, 2003 [93]	Hungary	> 6	50	9	34	N	0	3 (AH), 28 (SC)
Eckman, 2008 [94]	United States	> 6	73	23	59	N	57	Not stated
Lee, 2014 [95]	Taiwan	> 6	40 (20 + ASST) (20 − ASST)	20	47.5 (70) (25)	N	5	Not stated
Ulambayar, 2017 [96]	Taiwan	> 6	125 (64 + ASST) (61 − ASST)	64	24.8 (32.8) (16.4)	N	3.1	5 (AH)
Baioumy, 2018 [97]	Egypt	> 8	40 (18 + ASST) (22 − ASST)	40	52.5 (83.3) (27.3)	N	2.5	5 (AH)
Schoepke, 2019 [98]	Multinational	> 6	182 (107 + ASST)	0	58	N	–	3 (AH), 28 (SC)
Asero, 2020 [99]	Italy	> 6	20	20	50	N	–	Not stated

*AH* antihistamine, *SC* systemic corticosteroids

**Table 5:** Summary of studies detecting IgG anti-IgE antibodies in subjects with CSU and healthy controls

Reference (author, year)	Region	Definition of CSU in weeks	Number of CSU patients undergoing testing	Number of healthy controls undergoing testing	% prevalence of IgG anti-IgE autoantibody in study group	Functional assay (F) or non-functional assay (N)	% prevalence of IgG anti-IgE autoantibody in control group	Number of antihistamine (AH) and/or systemic corticosteroid (SC)-free days
Gruber, 1988 [100]	United States	–	6	32	50	N	0	Not stated
Fiebiger, 1995 [11]	Austria	> 8	32	15	69	N	26	Not stated
Tong, 1997 [101]	United States	> 12	50	20	12	F	5	2 (AH)
Eckman, 2008 [94]	United States	> 6	73	23	45	N	30	Not stated
Rojanapremsuk, 2015 [102]	United States	> 6	438	–	33	N	–	Not stated

*AH* antihistamine, *SC* systemic corticosteroids

**Table 6:** Summary of studies detecting antithyroid peroxidase (anti-TPO) antibodies in subjects with CSU and healthy controls

Reference (author, year)	Region	Definition of CSU in weeks	Number of CSU patients undergoing testing	Number of healthy controls undergoing testing	% prevalence of anti-TPO antibody in study group	% prevalence of anti-TPO antibody in control group	Number of antihistamine (AH) and/or systemic corticosteroid (SC)-free days
Verneuil, 2004 [103]	France	–	45	30	17.8	3.3	Not stated
Gangemi, 2009 [104]	Italy	> 6	95	0	26.3	–	Not stated
Nuzzo, 2011 [105]	Italy	> 6	54	108	22.2	1.9	Not allowed
Alpay, 2013 [106]	Turkey	> 6	50	50	12	4	3 (AH)
Cho, 2013 [107]	United States	> 6	27	20	11.1	20.0	Not stated
Wan, 2013 [108]	Taiwan	> 6	60	40	8.3	0	Allowed (AH)
Yadav, 2013 [109]	India	> 6	80	0	17.5	–	3 (AH), 21 (SC)
Kim, 2016 [110]	Korea	> 6	184	0	13.6	–	Not stated
Czarnecka-Operacz, 2017 [111]	Poland	> 6	145	35	15.9	–	Not stated
Kasumagic-Halilovic, 2017 [112]	Bosnia and Herzegovina	> 6	70	70	30	1.4	Not stated
Schoepke, 2019 [98]	Multinational	> 6	182	0	20.9	–	3 (AH), 28 (SC)

*Anti-TPO* antithyroid peroxidase, *AH* antihistamine, *SC* systemic corticosteroids

**Fig. 5:**
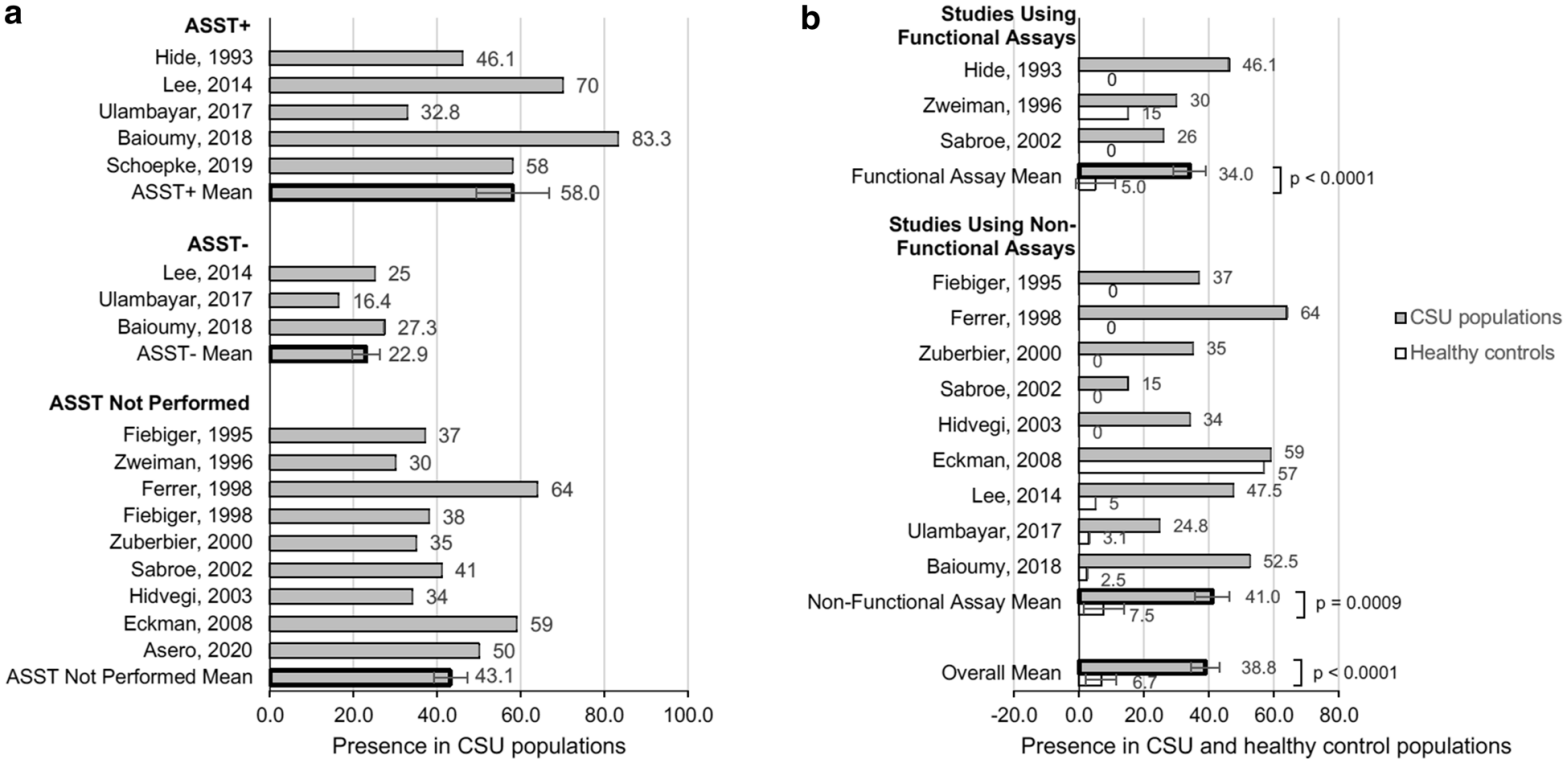
**a** Presence of IgG autoantibody to FcεR1α in CSU populations with and without ASST+. **b** Presence of IgG autoantibody to FcεR1α in CSU populations in 11 studies, compared to healthy control groups (p < 0.0001)

**Fig. 6:**
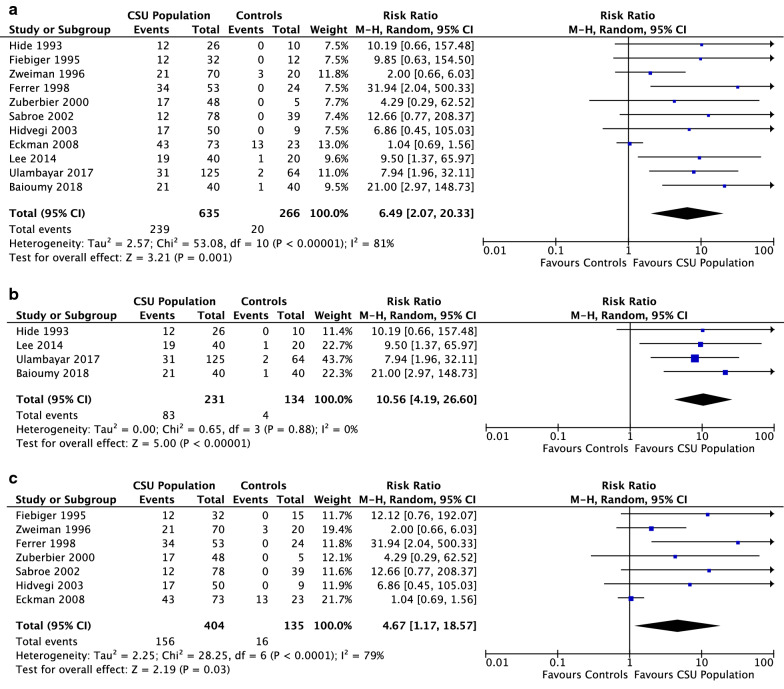
**a** Forest plot graphic assessing the relative risk of the presence of IgG Autoantibody to FcεR1α in all studies. M-H, Mantel–Haenszel approach. **b** Forest plot graphic assessing the relative risk of the presence of IgG Autoantibody to FcεR1α in studies that performed the ASST. M–H, Mantel–Haenszel approach. **c** Forest plot graphic assessing the relative risk of the presence of IgG Autoantibody to FcεR1α in studies that did not perform the ASST. M–H, Mantel–Haenszel approach

14 studies assessed the presence of IgG autoantibody directed to FcεR1α in CSU subjects. In nine studies that did not perform autologous serum skin tests (ASST), IgG anti-FcεR1α autoantibody was detected in 43.1% (SEM 3.4) of CSU subjects (Fig. 5a). In five papers that studied positive ASST CSU subjects, 58% of the subjects (SEM 8.8) had IgG anti-FcεR1α autoantibody, while in 3 papers that studied negative ASST CSU subjects, the autoantibody was detected in 22.9% of subjects. 11 of the 14 total studies that investigated the association of CSU with IgG anti-FcεR1α reactivity included a healthy control population with no history of atopy. On average, 38.8% of the CSU population (SEM 4.3) had IgG autoantibody directed to FcεR1α while only 6.7% of the healthy control group (SEM 4.7) had IgG anti-FcεR1α present (p < 0.0001) (Fig. 5b). In three controlled studies that used a functional assay to detect IgG anti-FcεR1α in CSU subjects, 34% of the CSU subjects had the autoantibody (SEM 5.4) compared to a 5% presence within control populations. The Hide 1993 study was the only study to include only ASST + CSU subjects. In nine controlled studies that used a non-functional assay, IgG anti-FcεR1α was detected in 41% of CSU subjects, while only 7.5% of the control group had the autoantibody present. In the Lee 2014, Ulambayar 2017 and Baioumy 2018 studies, the values from all CSU subjects (ASST positive and negative), were compared to the controls, in whom no ASST was performed. Combined RR was 6.5 (95% CI 2.1–20.3) with significant heterogeneity (p = 0.001; I^2^ = 81%), as shown in Fig. 6a. In four studies that performed ASSTs on CSU subjects, the combined RR was 10.6 (95% CI 4.2–26.6) with no heterogeneity (p < 0.00001; I^2^ = 0%) (Fig. 6b). In comparison, in 7 studies that did not perform ASSTs on CSU subjects, the combined RR was 4.7 (95% CI 1.2–18.6) with significant heterogeneity (p = 0.03; I^2^ = 79%) (Fig. 6c). Overall, the final estimate from the forest plots revealed an increased risk of presence of IgG autoantibody to FcεR1α in subjects with CSU.

**Fig. 7:**
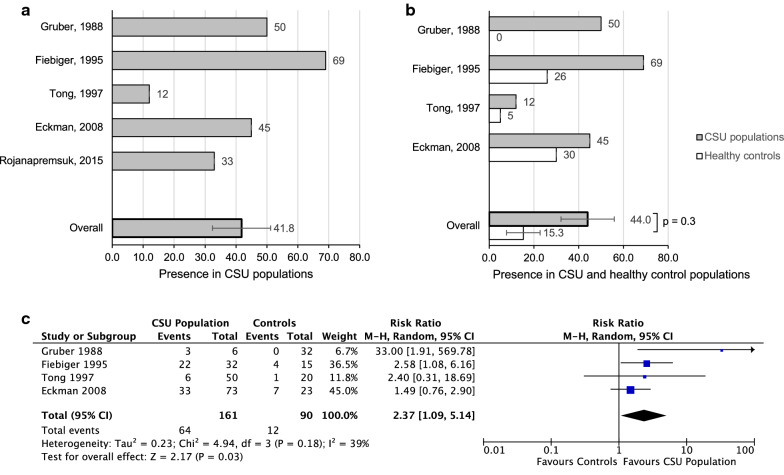
**a** Presence of with IgG anti-IgE antibody in CSU populations. **b** Presence of IgG anti-IgE antibody in CSU populations in 5 studies, compared to healthy control groups. **c** Forest plot graphic assessing the relative risk of the presence of IgG anti-IgE antibody in CSU populations compared to healthy controls. M–H, Mantel–Haenszel approach

The presence of IgG anti-IgE autoantibody in five CSU populations was 41.8% (SEM 9.4) (A). Four out of these five studies included a healthy control population with no history of atopy. 44% of the CSU groups (SEM 11.9) had IgG anti-IgE antibody, compared to only 15.3% in the healthy control groups (SEM 7.5) (p = 0.3) (Fig. 7b). Combined RR was 2.4 (95% CI 1.1–5.1), (p = 0.03, I^2^ = 39%), as shown in Fig. 7c.

**Fig. 8:**
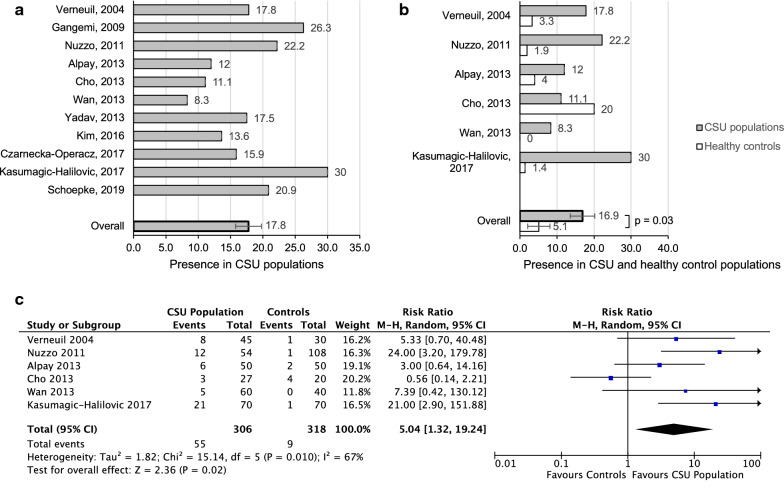
**a** Presence of anti-thyroid peroxidase antibody in CSU populations. **b** Presence of anti-thyroid peroxidase antibody in CSU populations in 11 studies, compared to healthy control groups. **c** Forest plot graphic assessing the relative risk of the presence of anti-thyroid peroxidase antibody in CSU populations compared to healthy controls, M–H, Mantel–Haenszel approach

The presence of anti-TPO antibody in 11 CSU groups was 17.8 (SEM 2.0) (Fig. 8a). Six out of these 11 studies included a healthy control population with no history of atopy. Anti-TPO antibody was detected in 16.9% of the CSU populations (SEM 3.3), compared to 5.1% in healthy control groups (SEM 3) (p = 0.03) (Fig. 8b). Combined RR was 5.0 (95% CI 1.3–19.2), (p = 0.02, I^2^ = 67%), as shown in Fig. 8c.

Assessing the total blood eosinophil percentage and/or absolute blood eosinophil count in subjects with CSU, from 608 publications retrieved from database searches following duplicate removal, 51 were screened for full-text review, which includes 5 papers from citation analysis (Table 7). Of these, 24 did not meet the outlined eligibility criteria, yielding 27 full texts from 13 countries that were included for analysis (Fig. 1d). 15 studies included information regarding the discontinuation of systemic corticosteroids prior to the commencement of the trial. One study, Anuradha et al. stopped systemic corticosteroids for 4 weeks and the mean eosinophil % was 4.2% (n = 60), while the median was 364 $\times$ 10^6^/L [23]. Another study, Dakhale et al., stopped systemic corticosteroids for 8 weeks and the mean eosinophil % was 6.8% (n = 64) [24]. The absolute eosinophil count was not provided. Gomez-Vera et al., excluded all patients who responded to normal dose antihistamines and had a history of asthma or allergic rhinitis, anaphylactic reactions, and patients using systemic or topical corticosteroids but only reported the median eosinophil count 0.170 × 10^3^/μL (IQR 0.100–0.300) [25]. Chang et al. didn’t state if subjects had taken corticosteroids or not and only included CSU subjects 1 to 18 years old (17/60 under 7 years old). In the 54 CSU subjects with lab work available, 13% (7/54) had elevated eosinophils [26].

**Table 7:** Summary of studies measuring blood eosinophil % and absolute eosinophil count in CSU subjects

Reference (author, year)	Country	Definition of CSU in weeks	Treatment	Number of CSU patients undergoing eosinophil testing	Mean blood eosinophil % (SD)	Median blood eosinophil % (IQR)	Mean absolute eosinophil count	Median absolute eosinophil count (IQR)	Number of antihistamine (AH) and/or systemic corticosteroid (SC)-free days
Toyoda, 1996 [113]	United States	–	None stated	30	–	–	164.3 (SE 54.6)	–	28 (SC)
Di Lorenzo, 1996 [114]	Italy	> 8–12	None stated	13	–	–	270 (SD 90)	–	2 (AH), SC not allowed
Kim, 1997 [115]	Korea	–	None stated	285	–	–	184.4 (SE 8.4)	–	28 (SC)
Grattan, 2003 [116]	United Kingdom	–	Loratadine & Prednisolone	7	–	–	121 (SE 13)	–	7 (AH), 28 (SC)
Garmendia, 2004 [117]	Venezuela	> 6	Ceterizine & Loratadine	32	–	–	146 (SD 132)	–	5 (AH), 3 (SC)
Anuradha, 2010 [23]	India	> 6	Loratadine	30	4.1 (1.6)	–	357 (SD 176)	–	3 (AH), 28 (SC)
			Levocetirizine	30	4.2 (1.4)	–	368 (SD 131)	–	3 (AH), 28 (SC)
Boonpiyathad, 2014 [118]	Thailand	> 6	Vitamin D	60	–	–	127 (SD 50)	–	AH allowed
Dakhale, 2014 [24]	India	> 6	Cetirizine	31	6.9 (1.9)	–	–	–	7 (AH), 56 (SC)
			Rupatadine	33	6.7 (1.5)	–	–	–	7 (AH), 56 (SC)
Woo, 2015 [119]	Korea	> 6	None stated	72	2.6 (1.8)	–	202 (SD 239)	–	Not stated
Dakhale, 2016 [50]	India	> 6	Rupatadine	30	6.9 (1.8)	–	–	–	7 (AH), 56 (SC)
			Olopatadine	30	6.8 (1.6)	–	–	–	7 (AH), 56 (SC)
Gomez-Vera, 2016 [25]	Mexico	> 6	AH, Omalizumab	76	–	–	–	170 (100–300)	Not allowed
Wardhana, 2017 [120]	Bali	> 6	None stated	25	–	–	176 (SD 120)	–	Not stated
Aitella, 2018 [121]	Italy	> 6	None stated	50	–	–	199 (SD 100)	190 (110–268)	7 (AH)
Cildag, 2018 [122]	Turkey	> 6	Omalizumab	41	–	–	–	160 (80–245)	Not stated
Türk, 2018 [87]	Turkey	> 6	Omalizumab	25	–	1.7 (0.9-2.8)	–	130 (79–195)	AH, SC allowed
Acer, 2019 [123]	Turkey	> 6	Omalizumab	106	–	–	150 (SD 170)	–	Not stated
Akdogan, 2019 [124]	Turkey	> 6	Omalizumab	74	1.9 (1.9)	–	–	–	Not stated
Kolkhir, 2019 [125]	Germany	> 6	None stated	1613	–	–	–	150 (80–230)	Allowed
Nazik, 2019 [126]	Turkey	–	Omalizumab	136	2.1 (2.0)	–	–	–	AH, SC allowed
Oliver, 2019 [127]	United States	> 6	None stated	23	2.5 (0.4)	–	–	–	AH allowed, SC not allowed
Tamer, 2020 [128]	Turkey	> 6	Omalizumab	60	2.5 (3.0)	–	250 (SD 89)	–	SC not allowed
Pediatrics studies									
Jirapongsananuruk, 2010 [129]	Thailand	> 6	None stated	94	–	–	256	–	7 (AH)
Chang, 2013 [26]	Taiwan	> 6	None stated	54	–	2.0	–	–	Not stated
Chansakulporn, 2014 [130]	Thailand	> 6	None stated	92	–	–	–	259	7 (AH)
Cavkaytar, 2015 [131]	Turkey	> 6	None stated	68	–	1.4 (0.8–2.2)	–	100 (100–200)	8 (AH)
Uysal, 2016 [132]	Turkey	> 6	Desloratadine	92	–	–	–	180 (80–290)	Allowed
Lee, 2017 [133]	Korea	> 6	None stated	57	3.1 (1.6)	–	–	–	Not stated

*IQR* interquartile range, *SC* systemic corticosteroid, *SE* standard error, *SD* standard deviation

**Fig. 9:**
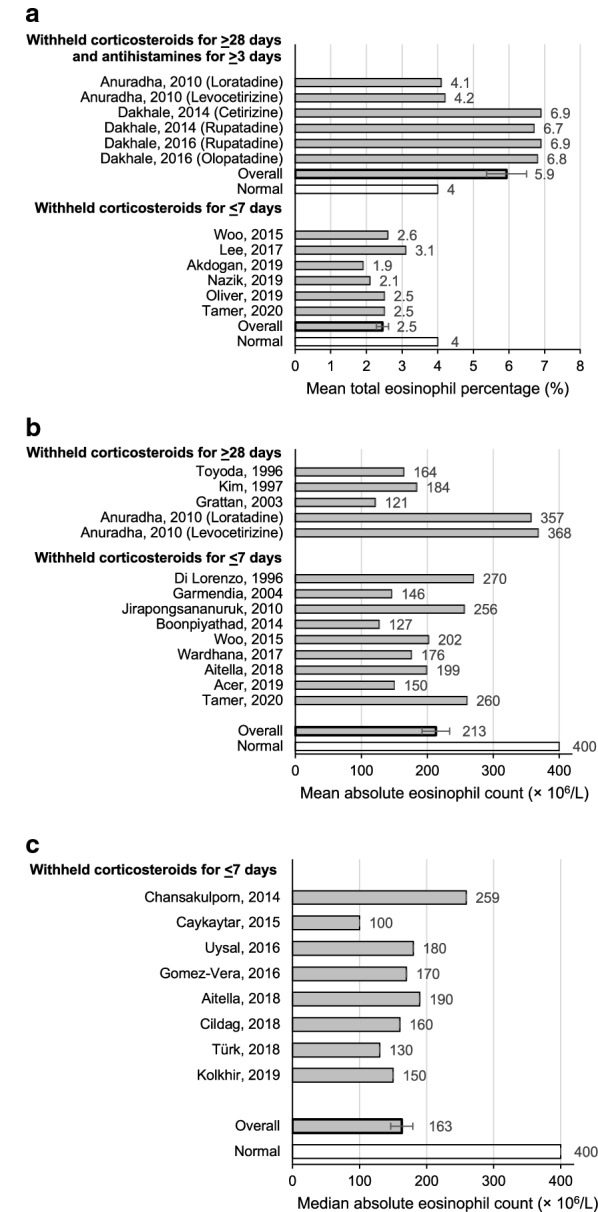
**a** Mean total eosinophil % in CSU populations undergoing treatment with omalizumab or H1-antihistamines that withheld corticosteroids for 28 days or longer and antihistamines for at least 3 days compared to studies that withheld corticosteroids for at least 7 days. **b** Mean absolute eosinophil count in CSU populations undergoing treatment with omalizumab or H1-antihistamines from studies that withheld systemic corticosteroids for 28 days or longer compared to studies that withheld systemic corticosteroids for less than 7 days. **c** Median absolute eosinophil count in CSU populations undergoing treatment with omalizumab or H1-antihistamines that withheld corticosteroids for less than 7 days

Combining the nine studies that assessed mean eosinophil counts in CSU subjects, the mean blood eosinophil percentage was 4.2% (SEM 0.6), which is similar to the normal of 4% (Fig. 9a). However, there were a large number of studies that measured eosinophils but allowed systemic corticosteroids (SC) and/or antihistamines (AH), which could have resulted in reduced counts. In three studies that withheld SC for ≥ 28 days and AH for ≥ 3 days prior to testing, the mean blood eosinophil percentage was 5.9% (SEM 0.6), compared to 2.5% (SEM 0.2) in six studies that only withheld SC for ≤ 7 days. Four weeks of treatment with loratadine resulted in 10% reduction while a 26% reduction was measured with cetirizine. Dakhale found a 16% reduction with 6 weeks of cetirizine and 31% reduction with 6 weeks treatment with rupatadine.

13 studies that evaluated mean absolute blood eosinophil counts in CSU subjects reported an average mean count of 213 $\times 10^{6} /$ L (SEM 21.0) (Fig. 9b). In four studies that withheld SC for 28 or more days prior to testing, the average mean absolute eosinophil count was 239 $\times 10^{6} /$ L (SEM 51.5). Conversely, in 9 studies that either allowed corticosteroids or did not define the length of time corticosteroid use was stopped for prior to testing, the average mean count was 198 $\times 10^{6} /$ L (SEM 17.8). Finally, eight studies reported an overall average median absolute blood eosinophil count of 163 $\times 10^{6} /$ L (SEM 16.6) (Fig. 9c).

**Fig. 10:**
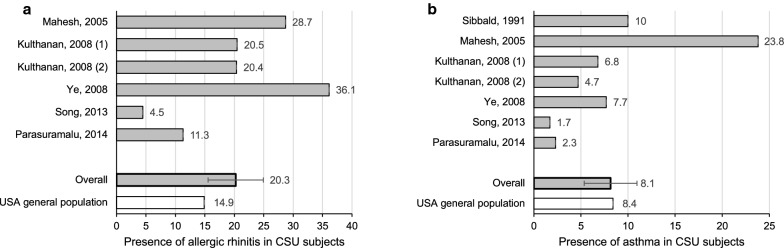
**a** Presence of allergic rhinitis in CSU subjects compared to the USA general population. **b** Presence of asthma in CSU subjects compared to the USA general population

The prevalence of positive personal history of other atopic diseases was identified in seven studies assessing positive SPTs to aeroallergens in patients with CSU, with AR (20.3%) (SEM 4.7) and asthma (8.1%) (SEM 2.8) being the most common (Fig. 10a, b). This is greater than the prevalence of AR (14.9%) [27], but not asthma (8.4%) [28] in the USA general population.

## Discussion

This is the first systematic review of the evaluation of T2 markers of allergic inflammation in CSU subjects.

An elevated tIgE is defined as above 137 kU/L, which represents the upper 90^th^ percentile in a general population [22]. CSU subjects had significantly elevated mean tIgE levels in all studies, with an average of 238 kU/L. Higher levels of tIgE in CSU subjects may indicate the presence of specific IgE to allergens, including aeroallergens.

We found the presence of positive SPT results to single or multiple aeroallergens in subjects with CSU was common, ranging from 2.3 to 67.9% in 21 studies. The ratio of multiple positive aeroallergen SPT in the CSU subjects was on average, considerably higher than the control groups (34.2% compared to 13.6%, p = 0.047), respectively. CSU subjects were 3.1 times more likely to have a positive aeroallergen SPT (95% CI 1.7–5.8, p = 0.0002) and 6.1 times more likely to have a positive perennial allergen HDM SPT (95% CI 3.7–9.9, p < 0.00001) than controls. In all 18 studies, an average of 1 in 4 CSU subjects had a positive SPT to HDM. Of the four studies that included control populations, approximately 1 in 4 CSU subjects had a positive SPT to HDM, compared to only 1 in 50 controls. As specific levels of IgE can increase with inhalant allergen exposure, SPT is important in identifying IgE sensitization to specific antigens. There are various treatment options that reduce aeroallergen specific IgE production, thus decreasing the likelihood of stimulating antibodies to IgE and its receptor. For instance, HDM sublingual immunotherapy treatment may act to effectively reduce HDM rhinitis and asthma [29]. Additionally, there is some evidence that allergen-specific immunotherapy [30] as well as the use of inhaled corticosteroids [31] promotes a long-term reduction of IgE levels to specific antigens and subsequent alleviation of symptoms.

The presence of aeroallergen positive SPT in CSU subjects was common; however, sensitization was not present in all CSU subjects. Non-sedating anti-H1 antihistamines remain the mainstay of initial intervention for treatment of CSU. H1 antihistamines can block SPT responses and decrease skin reactivity for upwards of 5 days, leading to false negative results. Thus, it is possible the rate of positive SPTs is actually higher than reported. Only 8 studies clearly stated that they discontinued use of antihistamine medications prior to proceeding with skin prick testing and reported the number of days of temporary discontinuation; however, of these eight studies, four withheld antihistamines for 3 days or less, which may not have been sufficient. For those studies that did not indicate the number of antihistamine-free days, the risk for an increase in false negative tests is elevated.

While omalizumab can suppress the SPT response for up to 6 months [32], no study indicated the discontinuation of omalizumab in their trials; although this was unlikely to have been important in the studies reviewed.

Oral corticosteroids are also commonly used to treat CSU [2]. While short-term oral corticosteroids may not alter the results of SPT [33], the effects of long-term treatment are not known. In a recent study evaluating the efficacy of omalizumab in the treatment of 68 subjects with severe CSU, 45.6% of subjects were reported to have an open prescription for systemic glucocorticoids at the time of treatment initiation. All of these subjects had required treatment with H1-antihistamines and oral glucocorticoids at some stage of their disease [34]. Similar patient demographics have been noted in the ASTERIA I trials where 50% of patients with CSU had been previously treated with corticosteroids [35]. In another study, it was reported that 57.9% of CSU patients had required treatment with systemic steroids prior to initiating omalizumab treatment. All patients had a medication history of H1-antihistamines, with 15.8% of the study population taking four times the standard dose on study day 1.

Consequently, in the present review, there is a high possibility that a significant percentage of subjects with CSU were undergoing treatment with oral corticosteroids. However, an evaluation of the prevalence of AR (20.3%, normal 14.9%) and asthma (8.1%, normal 8.4%) in CSU subjects revealed no considerable difference to the normal. The use of antihistamines and immunosuppressant medications, such as prednisone and ciclosporine A may mask AR and asthma symptoms, contributing to an under-diagnosis of these atopic conditions in CSU. Inhaled and intranasal steroids may be safer, less expensive and more effective therapies for AR and asthma. Because symptoms are masked by various CSU treatments, perhaps this may be a factor as to why skin testing is not perceived as relevant. That being said, most IgE is bound to the high affinity receptor on the mast cell and skin testing can determine if this is present [36].

Underrecognized asthma and AR could be important when considering patient safety, particularly if systemic steroids are withdrawn, while the urticaria is controlled with omalizumab. Hence, it may be important to consider inhalant sensitization in these patients. In the Xolair Treatment Efficacy of Longer Duration in Chronic Idiopathic Urticaria (XTEND-CIU) study, two anaphylactic episodes were reported whereby both patients were admitted to the emergency department with wheezing. The first patient had a history of mild, intermittent asthma while the second patient had a history of AR [37]. Therefore, skin testing and clinical suspicion of AR and asthma in these patients with CSU accompanied by assessment for inhalant allergen sensitivity may be important in reducing the risk of adverse reactions, particularly as omalizumab may have a systemic steroid sparing effect.

It has been demonstrated that the rate of superantigen producing *Staphylococcus aureus* colonization in the nasal cavity of patients with perennial AR was significantly elevated at 22% compared to healthy controls (6.7%) [38]. Similarly, patients with CRSwNP are also very likely to have frequent *Staphylococcus aureus* colonization in the nasal passages, which can cross airway epithelial barriers and induce elevated inflammation severity and vascular permeability [39]. Recent literature suggests the elevated presence of specific IgE to SEB in AR and CRSwNP patients, which may amplify mucosal inflammation and initiate a type 2 allergic response [18]. As IgE to SE is common in CSU patients [40], if T2 activity was controlled, complications involving SE and IgE to SEB in the pathogenesis of airway inflammation would be reduced, possibly indirectly leading to a reduction in CSU symptoms.

**Fig. 11:**
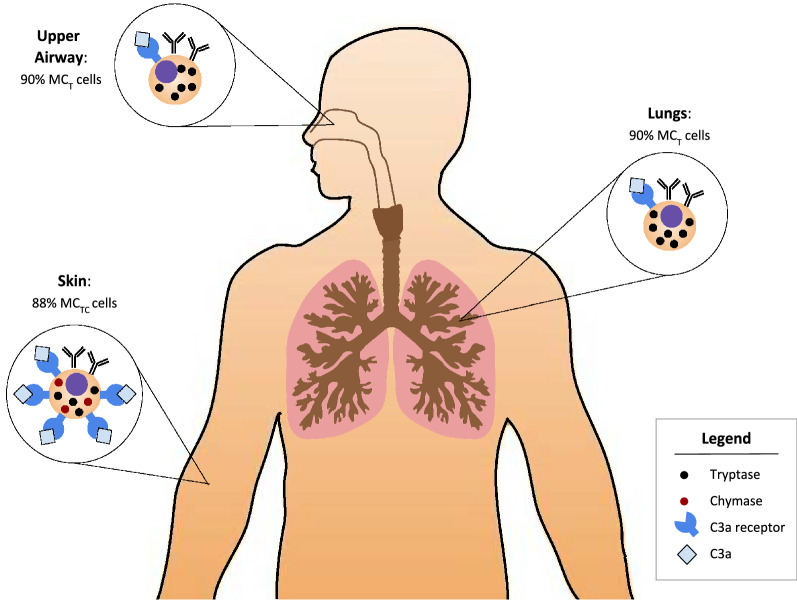
Chymase/tryptase mast cell type by tissue site

Approximately 90% of mast cells in dispersed lung tissue have been found to be of the MC_T_ (tryptase only) phenotype, while 88% of mast cells in dermal tissue were M_TC_ (tryptase and chymase) [41]. Airway epithelial cells, macrophages in lung tissue, and skin mast cells all produce anaphylatoxin C3a, which acts upon mast cells to cause degranulation [42, 43], and express its respective receptor, C3aR (Fig. 11). C3a and C5a complement factors are both upregulated in the lungs of subjects with allergic asthma [44]. While MC_TC_ cells found mainly in the skin are responsive to C3a, MC_T_ cells found mainly in the lung are not [45]. CSU may result from C3a being formed in areas of allergic inflammation such as the sinus, nose or lung. This may explain why CSU patients generally do not go on to have anaphylaxis, as C3a causes non-cytotoxic subacute degranulation of mast cells not through IgE cross-linking, and C5a has been found to upregulate the effect of IgG on cutaneous mast cells in CSU [46, 47].

In addition, thrombin can induce the generation of C5a in the absence of C3, bypassing the first stage of the complement cascade [48]. Recently, Zhu et al. outlined the concept of the interaction of the complement and coagulation systems in CSU [49]. They found increased levels of C5a and D-dimer in patients with active CSU, but C3 was not decreased [48].

Our search retrieved 4 studies that found increased mean absolute peripheral blood eosinophil counts in CSU subjects where systemic corticosteroids were withheld for at least 4 weeks compared to 9 studies that did not hold corticosteroids for 4 weeks or longer. Other studies that withheld corticosteroids for at least 4 weeks found a mean absolute eosinophil count of 239 $\times 10^{6} /$ L, which was not elevated. Where corticosteroids weren’t held, the mean eosinophil percentage, mean absolute eosinophil count and median absolute eosinophil count were not elevated. Studies did find that antihistamines reduced peripheral blood eosinophil counts in CSU subjects which may help explain these findings [23, 24, 50].

Three different anti-IL5 therapies given to patients with severe eosinophilic asthma and CSU have shown a benefit for symptoms of CSU. Mepolizumab was given to one patient, who experienced complete clearing of urticarial wheals 3 months after the first dose [14]. Subsequent discontinuation of mepolizumab prompted a relapse of CSU. Reslizumab was given to a patient with severe eosinophilic asthma, who had immediate improvement in her urticaria following treatment [15] Finally, benralizumab was given to a patient with severe asthma and symptomatic dermographism; the dermographism was markedly improved after the first injection [16]. After 3 months, the results of provocation tests for dermographism were negative. Mepolizumab and benralizumab are currently under evaluation for CSU (ClinicalTrials.gov Identifiers: NCT03183024, NCT03494881, respectively). In addition, dupilumab has also been described to help CSU in a series of 6 patients - 4 with atopic dermatitis and 2 with atopic dermatitis and asthma. It is also undergoing clinical trials in CSU subjects (ClinicalTrials.gov Identifiers: NCT04180488, NCT03749135) [17]. This demonstrates that initiating treatment of T2 inflammation with anti-IL5 or an agent which blocks IL4 and IL13 for subjects with asthma and/or atopic dermatitis who also have CSU, CSU symptoms may be improved.

In analyzing the literature on the presence of anti FcεR1α, anti-IgE, anti-TPO autoantibodies in patients with CSU, we found that the detection of all three autoantibodies in CSU patients were all significantly elevated in comparison to that of controls. In particular, CSU subjects were 6.5 times more likely to have IgG autoantibody against FcεR1α (95% CI 2.1–20.3, p = 0.001), 2.4 times more likely to have IgG anti-IgE antibody (95% CI 1.1–5.1, p = 0.03) and 5 times more likely anti-TPO antibody (95% CI 1.3–19.2, p = 0.02) than controls. These findings may indicate a potential autoimmune basis for the disease; however, further investigation is required to clarify the clinical significance of the presence of such antibodies in CSU and its association with disease activity. It is not clear if they could cause subclinical or partial degranulation of cutaneous mast cells, or if they just occur with T2 inflammation.

In this review, we found 21 studies where aeroallergen SPT was performed on CSU subjects, only 4 studies of which included a control group. One limitation of this study was the lack of studies including a healthy control group when performing SPTs for multiple aeroallergens or HDM in CSU subjects and healthy control groups. In addition, most studies did not indicate whether or not the use of antihistamines and systemic corticosteroids were appropriately discontinued, which may have resulted in more false negative SPT results. These treatments also may have masked underlying atopic conditions and may have reduced blood eosinophil counts. Two studies [51, 52] excluded subjects with atopic conditions entirely, which could have produced lower positive SPT results to aeroallergens. However, excluding these studies had minimal effect on the results observed and thus, were included in this review. Few studies evaluated blood eosinophil levels in CSU subjects off systemic steroids and antihistamines, but those that did demonstrated elevated levels in blood eosinophil percentage, but other studies did not find elevated absolute counts.

## Conclusion

In conclusion, this review supports findings from numerous studies that CSU patients are more likely to have positive SPT for both aeroallergens and HDM specifically. This may point towards future therapeutic options in treating CSU. Elevated levels of aeroallergen sensitization, tIgE, and IgG autoantibody to FcεR1α in CSU subjects may indicate the importance of T2 inflammation in the pathogenesis of CSU. Further studies to determine whether or not specific allergen avoidance, desensitization or improved control of the mucosal allergic inflammation present in asthma and/or rhinitis has any benefit in the management of CSU in sensitized individuals may be warranted.

## Data Availability

All data generated or analysed during this study are included in this published article.

## Abbreviations

CSU: Chronic spontaneous urticaria
SPT: Skin prick test
ASST: Autologous serum skin test
IgE: Immunoglobulin E
IgG: Immunoglobulin G
FcεR1α: High-affinity receptor for the Fc region of immunoglobulin E
anti-TPO: Anti-thyroid peroxidase
HDM: House dust mite
SE: Staphylococcal enterotoxin
tIgE: Total serum IgE
CRSwNP: Chronic rhinosinusitis with nasal polyps
AR: Allergic rhinitis
